# Effects of Rate-Limiting Steps in Transcription Initiation on Genetic Filter Motifs

**DOI:** 10.1371/journal.pone.0070439

**Published:** 2013-08-05

**Authors:** Antti Häkkinen, Huy Tran, Olli Yli-Harja, Andre S. Ribeiro

**Affiliations:** 1 Department of Signal Processing, Tampere University of Technology, Tampere, Finland; 2 Institute for Systems Biology, Seattle, Washington, United States of America; Baylor College of Medicine, United States of America

## Abstract

The behavior of genetic motifs is determined not only by the gene-gene interactions, but also by the expression patterns of the constituent genes. Live single-molecule measurements have provided evidence that transcription initiation is a sequential process, whose kinetics plays a key role in the dynamics of mRNA and protein numbers. The extent to which it affects the behavior of cellular motifs is unknown. Here, we examine how the kinetics of transcription initiation affects the behavior of motifs performing filtering in amplitude and frequency domain. We find that the performance of each filter is degraded as transcript levels are lowered. This effect can be reduced by having a transcription process with more steps. In addition, we show that the kinetics of the stepwise transcription initiation process affects features such as filter cutoffs. These results constitute an assessment of the range of behaviors of genetic motifs as a function of the kinetics of transcription initiation, and thus will aid in tuning of synthetic motifs to attain specific characteristics without affecting their protein products.

## Introduction

Genes function in networks, whose building blocks are motifs of few genes. Several motifs have been identified, which perform a specific function in networks [1]. Examples include genetic switches, which can be used as memory circuits or for digital control of processes; oscillators, which can be used for time-keeping and synchronization; and genetic filters, which can be used for noise filtering and computation via genetic logic [1].

In addition to the gene-gene interactions, the behavior of a motif depends on the expression pattern of each constituent gene. Investigating this dependency is of relevance given recent evidence that both mean level and the cell to cell diversity in RNA and protein numbers vary between genes by several orders of magnitude [2]. For that, we need to use models that account for the nature of gene expression, since genes with low expression levels are abundant in bacteria [2], [3]. Such low numbers cause the dynamics of motifs to be poised with correlations and low copy number fluctuations.

Much effort has been made to characterize the processes of transcription and translation in bacteria. In vitro studies [4], [5] showed that transcription, the process by which RNA molecules are produced, is controlled mostly at the promoter region of the gene. Once the RNA polymerase reaches the transcription start site and forms the closed complex, it remains there until the open complex is complete. Following this, the polymerase can escape the promoter and elongate along the DNA sequence, according to which the RNA sequence will be assembled. Both in vitro and in vivo studies suggest that the closed and open complex formations are the lengthiest (rate-limiting) steps of the process of gene expression, along with protein folding and activation.

Recently, the intervals between transcription events in individual, live cells have been measured for two promoters, lac-ara-1 [6] and tetA [7]. These studies suggest that, under optimal conditions, there are two to three major rate-limiting steps, which occur during initiation, that control both mean rate and noise in RNA production. These steps durations were also shown to vary widely with induction level and environmental conditions [6], 7. In that sense, they are major regulators of the dynamics of mRNA production.

Since the duration of the rate-limiting steps in transcription is both sequence-dependent and regulated by activator and repressor molecules, these steps are both evolvable and adaptive to the environment [6]. Since in prokaryotes translation is coupled with transcription, these steps are likely also key regulators of protein numbers [8]. However, it remains unknown to what extent one can tune the behavior of genetic motifs by selecting specific kinetics of initiation of the constituent genes.

In this work, we study the behavior of stochastic genetic motifs, while varying the kinetics of transcription initiation of the constituent genes. Two motifs are considered: one performs filtering in the amplitude domain, and the other in the frequency domain. The response of the motifs is quantified for a wide range of transcriptional dynamics that are in accordance with measurements.

The results indicate that the dynamics of these two genetic motifs, while dependent of the gene-to-gene interactions, is also affected by the kinetics of transcription initiation of each component gene. This, in turn, suggests that it is possible to engineer synthetic circuits to be more robust or having higher plasticity than the present ones, by selecting for promoters with appropriate initiation kinetics.

## Methods

### Gene expression

We use the delayed stochastic modeling strategy [9], [10], which correctly accounts for the low copy number effects, that is, the fluctuations and correlations, of the interacting components, coupled with non-exponential waiting times. The results are quantified from Monte Carlo simulations of the reaction system, using SGN Sim [11].

To model gene expression we use the following set of reactions. The syntax 
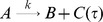
 denotes a reaction where 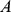 is transformed into 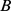 and 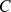, with a stochastic rate of 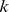. While 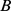 is released in the vessel of reactions instantaneously once the reaction occurs, 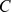 is released after a delay of 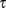 [10].
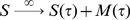
(1)

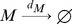
(2)

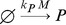
(3)

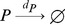
(4)where 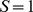 (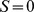) denotes that the promoter is free (occupied), 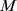 is the messenger RNA, and 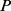 is the protein. Reaction 1 models transcription, Reaction 2 mRNA degradation (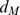 being the mRNA degradation rate), Reaction 3 translation (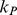 representing the per-mRNA translation rate), and Reaction 4 protein degradation (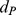 denoting the protein degradation rate).

The infinite rate set for Reaction 1 derives from the assumption that there is an inexhaustible pool of polymerases (which is a common assumption for bacteria in optimal growth conditions). The delay 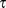 represents the effects of all rate-limiting steps, including the initiation of transcription up to the production of an mRNA. As mentioned, recent evidence suggests that, in *E. coli* under optimal growth conditions, 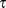 is determined to a great extent by the sum of two to three rate-limiting steps, each following an exponential distribution in duration [6], [7]. We use 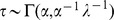, which denotes that the delay 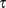 is drawn from gamma distribution with a shape of 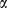 and a mean of 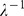. Integer values of 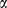 indicate that transcription consists of 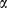 sequential steps, each with a rate of 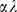. The gamma distribution has a coefficient of variation (the standard deviation over the mean) of 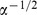 regardless of the mean (cf. unity of the exponential distribution, which is a gamma distribution with 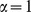). Consequently, values of 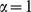 will result in a Poisson distributed 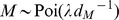, while values of 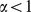 result in a more noisy (super-Poisson), and values of 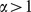 less noisy (sub-Poisson) mRNA number dynamics. We note that even if transcription initiation consists of sequential steps of unequal duration, the gamma distribution is still a good approximation. If the steps are of the same order of magnitude, they can be considered approximately equal, else, fast steps can be neglected.

Finally, we let 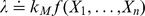, where 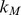 indicates the maximal expression rate of the promoter, and 
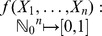
 is a regulatory function of the promoter, which depends on substances 
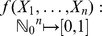
 through 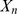. It is generally not known which steps are affected by which transcription factors, so we assume that each step is affected in an equivalent manner. The choice of these functions is discussed in the next section. Moreover, we let 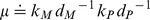, which coincides with the expected protein level of a gene under full expression.

Unless otherwise stated, we use the parameters 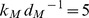, 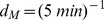, 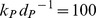, and 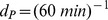. These values were selected in accordance with measurements in live *E. coli* [2]. In the results presented, each simulation is ran for 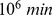, and the system is sampled uniformly every 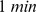. To assess the kinetics of initiation within a realistic range of parameter values, we set the number of rate-limiting steps 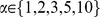. The first three have been observed in measurements of mRNA production kinetics in live *E. coli* cells [6], [7]. In vitro studies of the kinetics of this process (see e.g. [12]) provide evidence for the existence of, at least, five rate-limiting steps, namely, closed complex formation, three isomerization steps, and promoter clearance. We also study the effects of setting 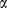 to 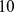 to observe the behavior of the model in limit conditions and due to the fact that some of the steps might be non-exponential in duration, thus requiring multiple exponentially distributed steps to be well described.

### Gene regulation

The genes are coupled by interactions between their promoter regions and the proteins they express. The activation/repression of a gene is achieved by the binding of the protein expressed by another gene. Once bound, this protein can either degrade while bound, or unbind. While bound, the propensity for the gene to express differs from the unbound case. The activation/repression of gene B by gene A could be represented by the following set of reactions:
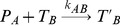
(5)

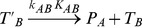
(6)

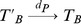
(7)where 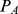 denotes the protein product of gene A, 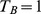 denotes that the binding site of the gene B for that protein is free, and 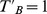 (implying 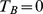) that the binding site is occupied. Here, Reaction 5 models the binding of the activator/repressor molecule 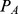 to the promoter region of gene B, Reaction 6 its unbinding, and Reaction 7 the degradation of a bound protein. The rate of binding is denoted by 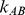 and the disassociation constant by 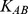.

To simplify the model, we take the limit 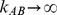. In this limit, the binding of the regulatory proteins is assumed to be much faster than the rate of transcription. It can be found that in this limit, the expectation 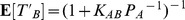 if 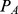 is constant. Following this, to implement the regulation, we vary the transcription rate such that:
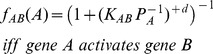
(8)

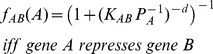
(9)and
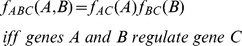
(10)where 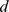 denotes the Hill coefficient, which represents the cooperativity of binding, (e.g. 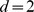 can be taken that there are two binding sites for a same type of protein) determining how steep the transition between on- and off-states (e.g. 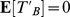 and 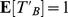) is. Also, the role of the disassociation constant in this context is now apparent, namely, it follows that 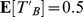 iff 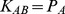. In our simulations, we use 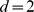, since many proteins are known to function in a dimeric form [13].

## Results

### Amplitude filtering

**Figure 1:**
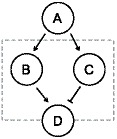
Illustration of the biphasic amplitude filter motif. In the biphasic amplitude filter, gene A acts as input to the filter, while genes C and D compose the filter, represented by the dashed box, along with the regulatory connections between each gene. The protein level of gene D acts as the output.

We start by examining how the properties of a genetic motif performing amplitude filtering are affected by the transcriptional dynamics. A genetic motif capable of behaving as a biphasic amplitude filter should allow the output to be active only for a certain range of input levels, which allows a process to be trigged by a narrow range of molecular concentration [1]. The region of inputs where the output is active is called the passband and the non-active regions are referred by stopbands. We model a biphasic amplitude filter consisting of four genes as follows. Gene A activates the expression of genes B and C, and gene B activates the expression of gene D, while gene C represses gene D. We model explicitly the expression of genes B through D, while the relative expression level of gene A acts as an input parameter. This is illustrated in Figure 1. Such a circuit was used to explain the narrow range of induction triggering the expression of Xbra in *Xenopus laevis* [14].

We simulate the model for various values of shape 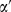 and rate 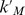 of transcription of genes B and C, while the output gene shape and rate are kept constant (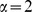, 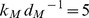). This is due to the fact that the effects of changes in 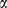 and 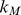 in the protein distribution of the output gene are more apparent and not related to the internal behavior of the filter, and because it allows the different cases to be easily compared. We set 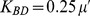 and 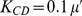, which is expected to produce a biphasic response (see Equations 11 through 13). In this, 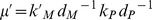 denotes the expression rate of genes B and C under full expression. To vary the mean input level, we vary the quantity 
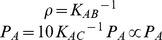
.

**Figure 2:**
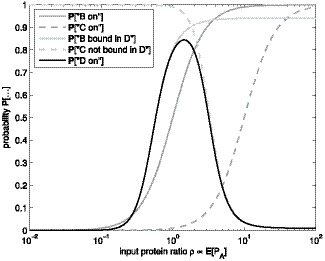
Event probabilities in biphasic amplitude filter. Probabilities of events in the biphasic amplitude filter as a function of the input protein level 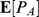. The solid black line denotes the probability that the output gene D is expressing, while the dark gray lines denote those of the intermediate genes (solid denoting gene B and dashed gene C). The probabilities that the intermediate genes allow the output gene to express are depicted by the light gray lines (solid denoting gene B and dashed gene C).

If all molecule numbers were constant, the response of the filter could be characterized by the following equations:
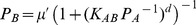
(11)

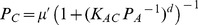
(12)

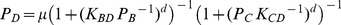
(13)which is a good approximation for high expression levels. Note that in Equation 13, 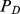 is a function of 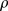, but invariant to the parameters 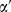 and 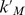, thus the effects of varying them lie beyond this formula. The response of the filter using Equations 11 through 13 is depicted in Figure 2.

The molecular levels will not be constant in our stochastic model. We quantify the noise in molecular levels using Fano factor (the variance over the mean), which is convenient, since Fano factor of Poisson-distributed molecules equals unity regardless of the mean. Even in the limit 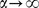 the protein levels will remain highly noisy (Fano factor 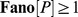), since in this case 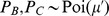 and their noise further propagates through the probabilistic expression of gene D to the output protein levels 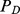.

**Figure 3:**
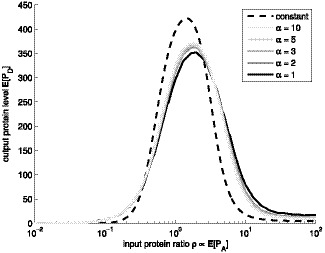
Mean response of biphasic amplitude filter. Mean response 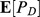 of the biphasic amplitude filter as a function of the protein level 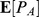 of the input gene, for various shapes 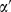. Different levels of gray denote different shape parameter 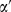. The simulations were performed with 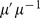 of 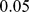. The dashed black line is an approximation, assuming constant molecular levels.

Next, we present the response of the biphasic amplitude filter using the stochastic model, and study how much it deviates from the expected response when the shape and rate of transcription are varied. The mean output level of the output gene D is presented in Figure 3. As expected, the response resembles the curves in Figure 2. Lower values of 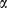 (which imply higher noise) produce slightly degraded performance in terms of the response of the filter. That is, the maximum output protein level will be lower, and the transition between the on- and off-states will be less steep. In addition, the increased noise makes the passband to shift toward a higher input level, since the distributions resulting from the model tend to have right skew.

**Figure 4:**
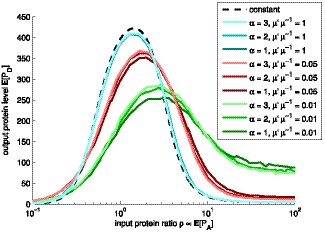
Mean response of biphasic amplitude filter for various transcription rates. Mean response 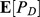 of the biphasic amplitude filter as a function of the input gene protein level 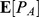, for various shapes 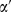 and rates 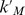 of transcription. Different levels of brightness denote different shape parameter 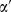. The simulations were performed with 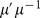 of 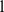 (cyan), 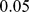 (red), and 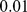 (green), in the order of decreasing performance. The three cyan lines overlap. We also performed simulations with 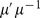 of 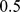, 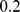, 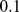, and 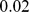 (not shown) to assert that the changes are generally nonlinear and more drastic for low mean levels. The dashed black line is an approximation, assuming constant molecular levels.

We also assessed the response for various mean expression levels 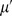 of the component genes (Figure 4). The results are qualitatively similar to those in Figure 3. Decreasing 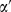 or 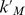 (either leading to higher noise) will degrade the filter performance. Moreover, as the expression rate is lowered the shape of the transcription takes greater role in determining the filter behavior. This implies that for rarely expressed genes, it might be important to have sub-Poissonian transcript dynamics, to compensate the increased low copy number noise.

Adding noise in the processes within the filter must shift downwards the value of the maximum output protein level. Generally, adding noise results in a flatter response, which can be interpreted as a degradation in performance, since the filter aims to selectively turn the output on or off. Furthermore, it is possible that adding noise also shifts the input level for which the maximal output is attained or the locations of the transition bands. The results depend on whether the input distributions and the response function of the filter are symmetric or not.

Finally, we assessed quantitatively the effects on the output of having different values of 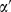, for each expression ratio of the input gene shown in Figure 4. For 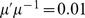, increasing 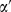 from 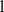 to 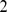, causes the output amplitude in the passband to increase by 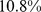. Increasing 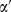 from 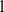 to 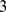 causes the output amplitude to increase by 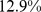. For other values of 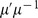, the differences are smaller. For example, for 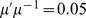, these increases are, respectively, 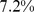 and 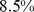, while for 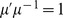, these differences are of the order of 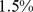.

**Figure 5:**
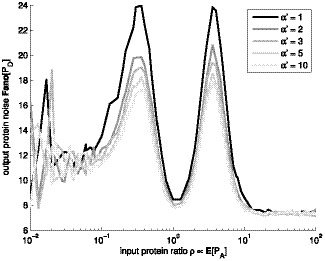
Noise of response of biphasic amplitude filter. Noise of the response 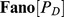 of the biphasic amplitude filter as a function of the input gene protein level 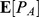, for various shapes 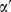. Different levels of gray denote different shape parameter 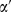. The simulations were performed with 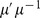 of 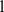.

Since our model dynamics is poised with noise, we study the noise in the output gene protein level, as a function of the input gene level. One might expect the noise to take a shape that is characteristic to the output gene, e.g. constant for Poisson, or some monotonically decreasing curve in our case. In the presence of noisy molecular levels in the circuit, this is generally not true. The noise in the output of this motif is expected to be higher in the transition bands of the biphasic amplitude filter, with the magnitude more characteristic to the output gene in the pass- and stop-bands. An example from stochastic simulations is presented in Figure 5.

**Figure 6:**
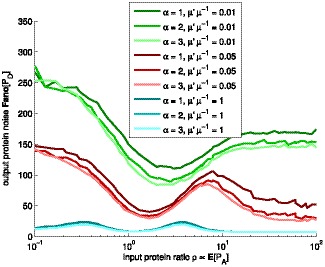
Noise of response of biphasic amplitude filter for various transcription rates. Noise of the response 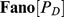 of the biphasic amplitude filter as a function of the input gene protein level 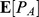, for various shapes 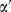 and rates 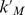 of transcription. Different levels of brightness denote different shape parameter 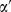. The simulations were performed with 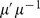 of 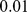 (green), 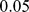 (red), and 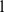 (cyan), in the order of decreasing noise. We also performed simulations with 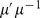 of 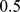, 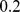, 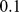, and 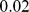 (not shown) to assert that the changes are generally nonlinear and more drastic with low mean levels. The dashed black line is an approximation, assuming constant molecular levels.

From Figure 5 we find that even when the effects of changes in transcription initiation on the response of the biphasic amplitude filter are slight, the change in the fluctuations of the protein numbers of the output gene might be significant. In Figure 6, we present the output noise for various mean levels. For very low expression levels, the low copy number noise in the output becomes dominant.

As a consequence of the amplification of the noise in the transition bands, the output of the filter becomes unpredictable in these regions. Therefore, for this circuit to operate properly in these regions, it is of importance to minimize the noise in the genes composing the filter, for example, by adding rate-limiting steps in initiation. Alternatively, regulation schemes that can provide steeper transition bands are required, which can be accomplished via regulatory schemes of higher-order. We hypothesize that the latter scheme has less effect, since it cannot remove the problem, only reduce its effects. Moreover, it is harder to implement in real genetic circuits, as it requires altering both the protein and the promoter sequences.

### Frequency filtering

In this section, we study the effects of changes in the transcription dynamics to a motif that performs filtering in the frequency domain. It is known that changes in the transcriptional dynamics can affect the period and its robustness of genetic oscillators [15], so we expect that these changes affect the response of certain frequency filters as well.

**Figure 7:**
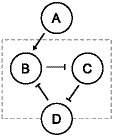
Illustration of the frequency filtering motif. In the frequency filtering motif, gene A acts as an input to the motif, while the filter consists of genes B, C, and D in a feedback loop structure along with the modulation by the input, represented by the dashed box. The protein level of gene D acts as an output of the filter.

We constructed a motif that can perform low-pass frequency filtering composed of four genes (A through D). This filter suppresses highly transient signals while letting slowly varying signals to pass through as-is. Such a filter would allow a specific set of genes to be subject to only stable signals, by filtering out fast fluctuations in the numbers of the regulatory molecules. Here, gene A acts as an input, required to enable the expression of gene B. Gene B represses gene C, C represses D, and D represses B, that is, genes C through D form a loop (three-gene repressilator). The structure of the motif is illustrated in Figure 7.

When a periodic signal 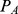 is applied, the behavior of this circuit should vary, depending on the frequency of the signal. When the signal is of high frequency, the feedback loop should be the main responsible for the frequency content of the output. For low frequencies, the input from gene A will disconnect the feedback loop periodically, and lower frequencies, including that of 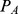, are introduced in the output. Thus, it is expected that the modulated circuit would have a synchronization point when the input frequency equals that of the repressilator, and that a phase transition would occur in the output frequency response.

For simplicity, we let the Hill coefficient 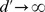, in the regulatory connection where A activates B. That is, the regulatory connection becomes Boolean, with a threshold of 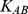. We denote the Boolean input signal by 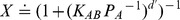. This allows us to omit the explicit modeling of gene A, and consequently this parameter does not need to be determined. Instead, we can apply an arbitrary 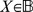. In this case, it does not matter if the connection is an activating (as in Figure 7) or repressing, since the Boolean input can be flipped.

First, we let the input signal to be constant 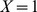. We analyze the periodic behavior characteristic to the submotif of genes B, C, and D. Since the genes B, C, and D are identical, we can treat them interchangeably and quantify the distribution of periods from any of the protein levels, denoted by 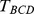, from the zeros of the autocorrelation function of each time series.

**Figure 8:**
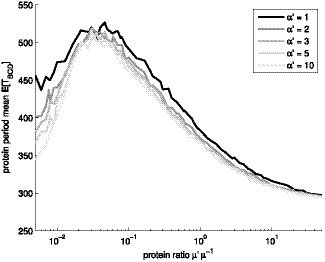
Mean period of frequency filtering motif with constant input. Mean period of the protein levels of genes B, C, and D (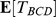), for constant input 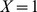. Different levels of gray denote different shape parameter 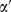.

We simulate our model for values of shape 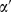 and rate 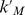 of genes B, C, and D, and 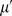 is defined analogously to the previous subsection. Moreover, the disassociation constants are set to 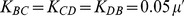, which were found to produce an oscillatory signal under constant input. The mean period of the protein levels of genes B, C, and D, as a function of the mean expression level 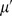 of the genes, is shown in Figure 8.

Interestingly, the period changes as a function of the number of steps in transcription initiation. Also, changing the mean transcription level affects the period (note that the disassociation constants are a function of the expected expression level 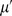, which would make a deterministic model invariant of 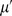).

**Figure 9:**
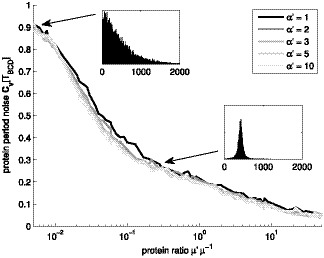
Noise in period of frequency filtering motif with constant input. Noise in the period of the protein levels of genes B, C, and D (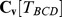), for constant input 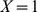. Different levels of gray denote different shape parameter 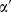. The insets exemplify the distributions of periods 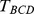 for shape of 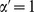 and ratios 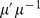 of 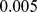 and 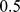 (units of the x-axis are seconds).

We also examined if the robustness of the period is affected. We quantify robustness by the coefficient of variation of the periods of the protein numbers. This measure is convenient, since it equals unity for exponentially distributed periods regardless of the mean. The results are shown in Figure 9. For low mean protein numbers, the period becomes unpredictable (i.e. exponential-like), whereas for moderate levels, the period distribution is Gaussian-like, due to lower noise in transcript production, implying more robust period length. The shapes of the distribution were verified from period histograms (see examples in the insets in Figure 9).

**Figure 10:**
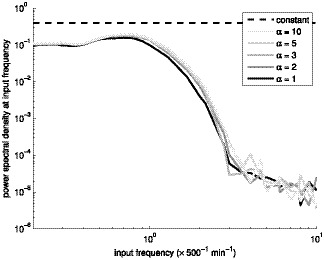
Power spectral density of the frequency filtering motif. Power spectral density of the frequency filter as a function of the input frequency. Different levels of gray denote different shape parameter 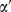. The simulations were performed with 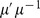 of 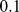. The dashed black line represents the PSD of the input 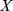 at the input frequency.

Next, we apply an unbiased Boolean square wave to 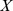, that is, 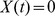 for time 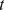 that satisfies 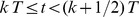 with any integer 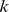 and 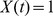 otherwise, and we denote its frequency by 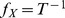, where 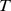 refers to the period. The autocorrelation function of this signal 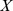 is a triangular wave of the same frequency, and consequently its spectral power is concentrated to the harmonics of 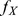. The spectral power is measured in terms of power spectral density (PSD), which is given by the Fourier transform of the autocorrelation function and measures how much of the signal power per unit frequency is concentrated around certain frequency. Specifically, the PSD of 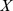 at frequency 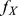 is 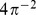 (cf. Figure 10).

We measure the power spectral densities of the input 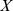 and the output 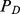. An example is shown in Figure 10, with the input PSD plotted for reference. The motif exhibits a low-pass behavior in the frequency domain. Frequencies lower than those corresponding to the mean period of the three-gene submotif when functioning independently (see Figure 8) are only slightly attenuated (amplification factor of 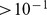). In contrast, higher frequencies are highly attenuated (amplification factor of 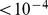).

**Figure 11:**
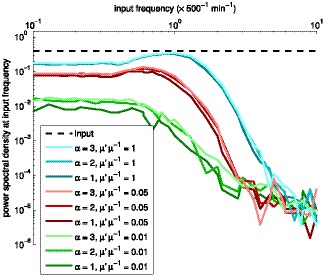
Power spectral density of the frequency filtering motif for various transcription rates. Power spectral density of the frequency filter as a function of the input frequency, for various shapes 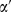 and rates 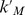 of transcription. Different levels of brightness denote different shape parameter 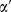. The simulations were performed with 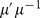 of 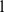 (cyan), 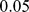 (red), and 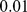 (green), in the order of decreasing performance. We also performed simulations with 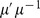 of 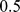, 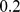, 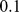, and 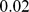 (not shown) to assert that the changes are generally nonlinear and more drastic with low mean levels. The dashed black line represents the PSD of the input 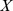 at the input frequency.

Changing the shape parameter 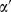 of the transcription results in slight variations in the performance of the frequency filter, while the main characteristics are not changed. Namely, the attenuation of the frequencies is of the same order of magnitude, more noisy shapes resulting in slightly higher attenuation in the passband. Moreover, the cutoff frequency is affected by changes in the characteristic frequency of the three-gene submotif (Figure 8). We also varied the transcription rate 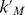 of the genes in the motif (Figure 11). Again, lower transcription rates, implying more noise in mRNA and protein levels, degrades performance, similarly to when varying 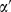. The changes in the steepness of the transition band of the filter are more apparent in the former case.

Similarly to the amplitude domain filter, the performance of the frequency domain filter is affected by changes in the transcriptional dynamics of the constituent genes. A transcription process that is less noisy results in a frequency filter with steeper transition bands. Consequently, an efficient frequency domain filter requires limited noise level in transcription, which in the case of low transcript levels can be implemented by a promoter with a sequential initiation process. Interestingly, the cutoff frequency of the filter is also affected by the kinetics of transcription.

As in the case of the amplitude filter, we assessed quantitatively the effects on the output of having different values of 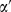, for each expression ratio of the input gene shown in Figure 11. For 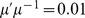, increasing 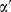 from 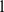 to 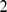, causes the magnitude of the PSD in the passband to increase by 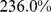. Increasing 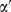 from 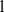 to 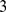, causes the PSD to increase by 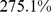. For other values of 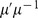, the differences are smaller as before. In particular, for 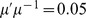, these increases are, respectively, 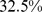 and 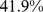, while for 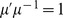, these differences are of the order of 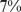.

## Discussion

Motivated by recent findings of the relevance of the kinetics of the process of transcription initiation on the dynamics of RNA production in bacteria [6], [16], we investigated the functioning of genetic filter motifs as a function of the kinetics of transcription initiation of the constituent genes. We focused on two common filters, namely, an amplitude filter and a frequency filter, as these have several practical applications. One major concern regarding their performance is that most genes in bacteria exhibit very low expression levels. We investigated whether one can utilize the multi-step nature of the process of initiation to compensate for the low copy number noise.

We found that, for realistic parameter values, genetic motifs with stochastic dynamics differ significantly from their deterministic counterparts. Consequently, the latter do not serve as a means to predict the realistic behavior of genetic motifs in live cells. Also, for low expression levels, high noise in the transcripts production significantly degrades the performance of the motifs. The effects of low copy number noise can be compensated by a multi-step (less noisy) transcription process. We suggest that natural motifs with low-expressing constituent genes might employ a multi-step transcription initiation process so as to limit the noise in the mRNA and protein levels, therefore allowing the motif to behave robustly.

The sequence-dependent distribution of transcripts production can have intriguing effects on the behavior of the motifs. These were most prominent in the characteristic frequency of the oscillatory circuit, in which, within a realistic interval of parameter values, it is possible to have a period double that of the one of high mean levels. Importantly, in both motifs studied, the cutoffs that separate the different regimes of operation of the filters were found to be tunable. The effects of changing the kinetics of transcription initiation were found to be slight, partly masked by the noise, but non-negligible.

It is known that changes in the kinetics of the sequential process of transcription initiation affect the dynamics of mRNA abundances of individual genes [16], [17]. Here, we provided tentative evidence that these changes affect the behavior of genetic motifs as well. This is of relevance, since both the number and the kinetics of these steps are dependent of the promoter sequence and transcription factors alone, i.e., are independent of the protein coding region. Due to this, we hypothesize that it is possible to alter the kinetics of a genetic circuit significantly by replacing the promoter region of the constituent genes, without the need of altering the protein under their control. Further, we hypothesize that changes in the promoter sequence of the constituent genes of motifs constitutes a significant degree of freedom in their evolutionary process in natural organisms.
